# Research Progress on Bioactive Substances of Beets and Their Functions

**DOI:** 10.3390/molecules29194756

**Published:** 2024-10-08

**Authors:** Chun Bian, Lanyang Ji, Wei Xu, Shirong Dong, Nan Pan

**Affiliations:** 1 College of Food Engineering, Harbin Institute, Harbin 150076, China; xweihappy@163.com (W.X.); dongshirong118@126.com (S.D.); pannan36@163.com (N.P.); 2 Heilongjiang Grain Quality Safety Monitoring and Technology Center, Harbin 150001, China; 2669911@126.com

**Keywords:** beets, phenolic substances, betalains, chemical components, function characteristics

## Abstract

As a globally cultivated and economic crop, beets are particularly important in the cane sugar and feed industries. Beet pigments are among the most important natural pigments, while various chemical components in beets display beneficial biological functions. Phenolic substances and betalains, as the main bioactive compounds, determine the functional characteristics of beets. This review categorizes the basic types of beets by the chemical composition of bioactive substances in their leaves, stems, and roots and emphatically summarizes the research progress made on the functions of two major substances in different types of beets: phenolic compounds and betalain pigments. This study provides useful insights for the comprehensive and effective application of beets in the health food and pharmaceutical industries.

## 1. Introduction

*Beta vulgaris* (L.) subsp. *maritima* (L.) *Arcang*, also known as sea beet, is a biennial herbaceous plant of the genus Chenopodiaceae. It is generally believed to have originated from the island countries along the Mediterranean coast and several countries in West Asia [1,2]. In ancient times, wild beets were consumed mainly for their leaves’ medicinal properties. In 2000 BC, the Greeks and Romans began domesticating wild beets. In 18th-century Europe, nutritious large-root beets (*Mangelwurzel*) were widely used to feed cattle. Commercial sugar production from beets began in 1747 when Andreas Marggraf from Germany first isolated sugar from beetroot. Since then, large-scale crossbreeding has been established, producing the various colors and shapes of beets [3,4]. Beets were introduced in China around the 8th century AD. They are warm-loving and cold-resistant, mainly grown in Xinjiang, Heilongjiang, and Inner Mongolia. Their cultivated subspecies are categorized into the following four groups: leaf, table, feed, and sugar beets [5,6,7].

**Figure 1:**
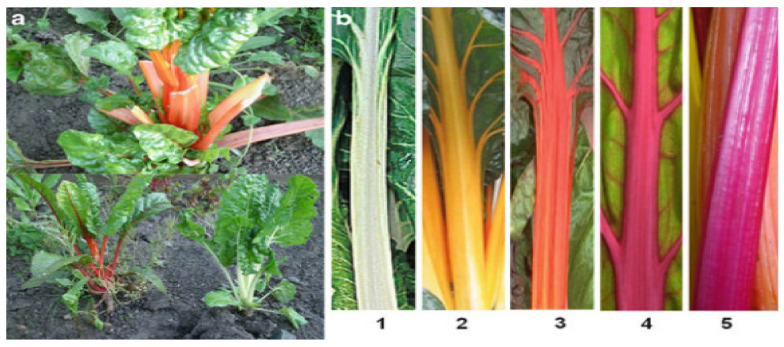
Leaf feet. (**a**) Field growth showing high diversity. (**b**) The effect of pigments on the petiole’s color. (1) A beet lacking a pigment; (2) dominant betaxanthin content; (3) high betaxanthin + low betacyanin contents; (4) low betaxanthin + high betacyanin contents; (5) dominant betacyanin content.

**Figure 2:**
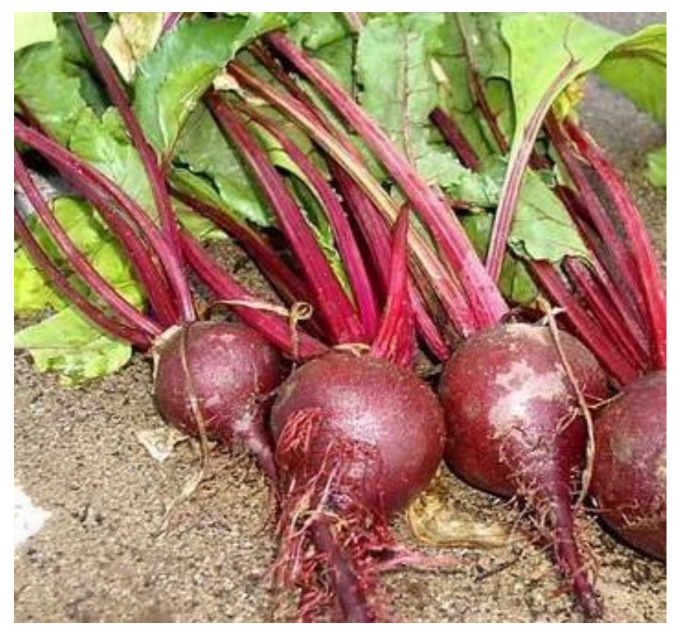
Table beet.

**Figure 3:**
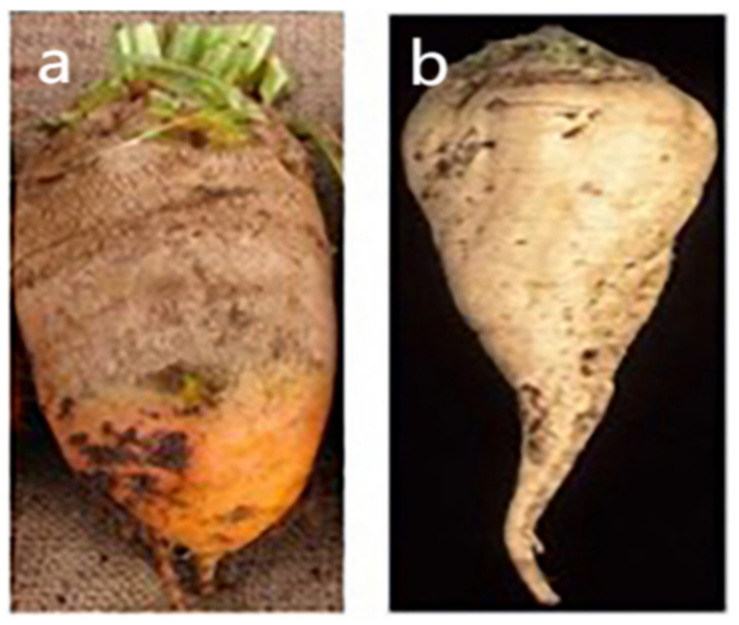
Fodder beet (**a**) and sugar beet (**b**).

Depending on the type, the leaves, petioles, and roots of beets may be edible or used as animal feed; the composition varies greatly between different types. The morphology of leaf beets is shown in Figure 1. Among all types of leaf beets, those with thicker petioles are known as Swiss chard, a domesticated species, while those with spinach-like petioles are wild *Beta maritima*, a primitive species. Leaf beets can be used both as an edible leafy vegetable and as an ornamental plant. The morphology of table beets, also known as beetroots or red beets, is shown in Figure 2. They are mainly consumed for their swollen root, which is the main source of the red pigment. Feed beets include two main types: mangel beets and fodder beets. The former has around a 13% lower dry matter content compared to the latter. Sugar beets are bred from fodder beets [5,6,7,8]. Like sugar cane, sugar beets are one of the two main raw materials in the sugar industry. They are economic crops widely planted in temperate regions. Figure 3 illustrates the morphologies of feed and sugar beets. In addition to providing specific micronutrients in the daily diet, beets also contain high concentrations of various bioactive compounds [9]. This paper summarizes the current status of research on the functions of bioactive substances found in different types of beets.

## 2. Phenolics

Beets contain phenolics, which are natural antioxidants and free radical scavengers. They exert beneficial effects on human health, including anti-inflammatory, anti-hypertensive, anti-tumor, and anti-cholesterol activities. Phenolics include phenolic acids, flavonoids, and phenolic aldehydes, and they are mostly found in the stems, leaves, and peel of beets. They are often removed as waste during processing or cooking [10,11]. In general, beet stems and leaves have relatively high contents of phenolics. The total phenolic content in the skin, crown, and flesh of beetroots decreases in this listed order [12]. Table 1 shows the phenolic content of different beet parts, dividing by phenol type and beet type.

### 2.1. Antioxidant Activity of Beets

Fernando et al. extracted polyphenols with high biological activity from the waste generated during red beetroot juice extraction using the ultrasound-assisted ethanol (30%) solution method [21]. In this study, a total of 12 different polyphenols, mainly including gallic acid, syringic acid, caffeic acid, and ferulic acid, were identified using HPLC-MS. Among them, seven were hydroxycinnamic acid derivatives, four were flavonoids, and one was trihydroxybenzoic acid. This result is consistent with the findings of Kujala et al. [22]. Liu et al. evaluated the polyphenol content and antioxidant capacity of eight types of red-stalked leaf beet and found that the polyphenol content was higher in old leaves compared to young leaves. Notably, the polyphenol content was higher in leaves than in petioles [23]. Moreover, this study showed that the polyphenol content is positively correlated with antioxidant activity. The DPPH radical scavenging activity of the red-stalked green-leaved type was significantly more potent than that of the red-stalked red-leaved type [23]. Liu et al. investigated the effect of harvest temperature on the phenolic composition and content in the leaves and stems of inbred types of *Lycium barbarum Miller* [23]. They found that their antioxidant activity was positively correlated with the phenolic content (total phenolic and total flavonoid contents). The stems of *Lycium barbarum Miller* displayed the strongest antioxidant activity at a relatively high temperature. Backes et al. extracted phenolics from beet stems and leaves with the heat-assisted ethanol aqueous solution method [24]. They measured the total phenolic content in the extract using the Folin–Ciocalteu method and expressed the reduction value of the extract as gallic acid-equivalent (GAE) milligrams per gram of the sample. This study revealed that both DPPH and ABTS in the extract had potent antioxidant capacity, and there was a positive correlation between the reduction value and ABTS value. Comparing these values indicated that the polyphenol reduction capacity of beet stem and leaf extracts is higher than that of oranges, strawberries, or general vegetables [24].

### 2.2. Tumor-Suppressive Functions of Beets

Cancer cells can escape from homeostasis through mutations in key signaling pathways associated with cell proliferation, apoptosis, and cell cycle control [25]. Cancer is one of the leading causes of morbidity and mortality in humans. Natural polyphenolic compounds, including those extracted from food raw materials, can prevent and control certain types of cancer and reduce their recurrence rates [26]. This provides an opportunity for adjuvant cancer therapy [18]. Furthermore, flavonoids in beet leaves have demonstrated inhibitory effects against various types of cancer cells.

Mancini et al. extracted phenolics from beet leaves and isolated apigenin from the extract [27]. In this study, prostate cancer cells (DU-145 and PC-3) were treated with this extract for 48 h. The results indicated that 100 ug/mL of the extract or 7.8 ug/mL of apigenin can significantly inhibit the proliferation of cancer cells [27]. Further studies demonstrated that this was achieved by apigenin’s effective regulation of mTOR pathway proteins, cell cycle, oxidative stress, and apoptosis-related proteins.

In a study by Appiah et al., the therapeutic effects of beets processed in various ways were evaluated using the Fisher 344 male rat model of colon cancer [28]. The results showed that the flavonoid and total phenolic contents of beets processed by freeze-drying were significantly higher than those processed by freezing or steaming and bleaching. Specifically, the freeze-dried beets contained 56.89 mg CE/100 g and 230.62 mg GAE/100 g. Furthermore, both flavonoids and phenolics displayed significant antioxidant activity and markedly reduced the incidence of azomethane (AOM)-induced abnormal crypt lesions (ACF) by up to 80%.

## 3. Betalains

**Figure 4:**
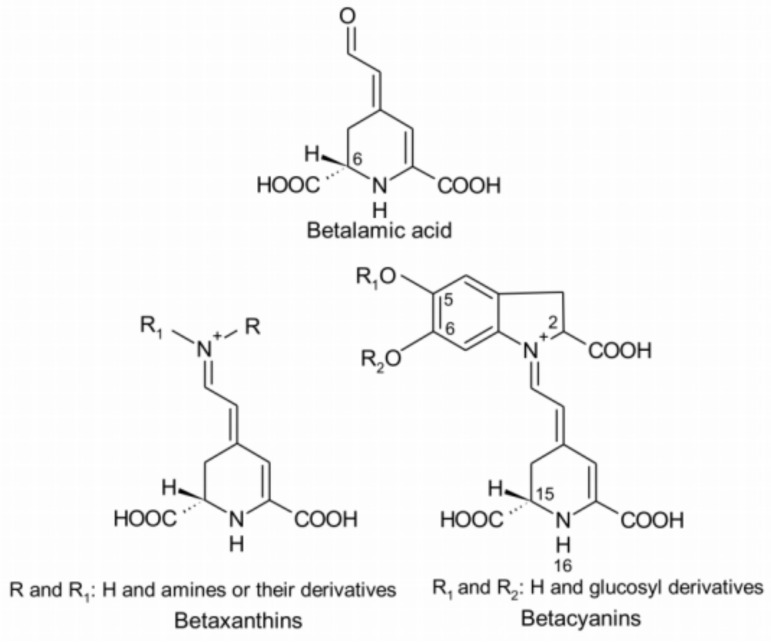
Resonance structures of betalamic acid, betaxanthins, and betalains [33].

**Figure 5:**
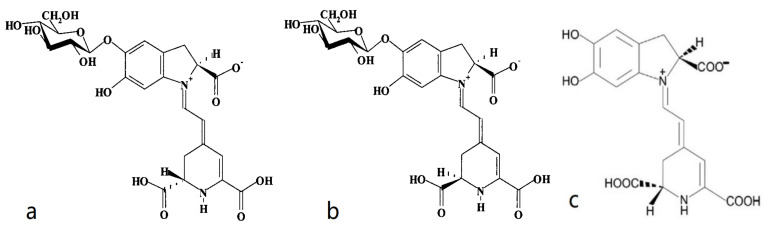
Resonance structures of several betacyanins [12,34]: (**a**) betanin, (**b**) isobetanin, and (**c**) betanidin.

The deep red color in red beetroots comes from high concentrations of betalains, which are secondary plant metabolites of phenolics. Early studies suggested that betalains are a type of anthocyanin [29], but later studies revealed significant structural differences between the two compounds. These findings indicated that the two are distinct natural pigments with unique metabolic pathways. Betalain is an imine derivative of betalamic acid. As shown in Figure 4, betalains can be categorized into red-purple betacyanins and yellow-orange betaxanthins based on their chemical structures. These compounds have been detected in purple-red, orange, and yellow plant species within closely related families of the order Caryophyllales, as well as in a few higher fungi. Betalains with 78 different structures are present in the flowers, fruits, and leaves of beetroots. They accumulate in cellular vacuoles, particularly in the epidermis and subcutaneous tissues [30,31], with the content decreasing in the following order: the epidermis, crown, and flesh of beetroots [12,22]. Betacyanins mainly include betanin, isobetanin, betanidin, isobetanidin, prebetanin, and neobetanin [12,30] (Figure 5). Betaxanthins include vulgaxanthin I and II [30,32].

Wruss et al. evaluated the composition of seven beetroot products and juices in Upper Austria [35]. This study found that the total betalain content ranged from 0.8 to 1.3 g/L (approximately 60% betacyanins and 40% betaxanthins), accounting for 70–100% of the total phenolic content. The other phenolic substances identified in the composition included hydroxycinnamic acid, which accounted for 2.6% of the total phenolics. Research by Pandita et al. revealed that fresh beets contain up to 200 mg of betanin and 450–500 mg of betalain per 100 g sample [36]. Studies using various cancer cells have shown that betalains display a potent chemopreventive effect, in addition to their role as natural colorants used in the food industry [35]. While potent radical-scavenging and antioxidant activities of betalains have been identified through in vitro experiments, in vivo model animal experiments and bioavailability studies suggest their beneficial role in the diet [37].

### 3.1. Free Radical-Scavenging and Antioxidant Effects

Antioxidants can scavenge free radicals and prevent the oxidation of biomolecules induced by reactive oxygen species, thereby helping to prevent associated diseases. Similar to polyphenols, the antioxidant potential of betalains can be assessed by measuring their ability to absorb harmful free radicals or oxygen radicals, as well as their ability to prevent certain oxidative stress-related diseases [38].

#### 3.1.1. In Vitro Antioxidant Activity of Betalains

In 1998, Escribano analyzed the free radical scavenging ability of betacyanins and betaxanthins extracted from beetroots [39]. To date, various studies have demonstrated the potent in vitro antioxidant activity of betalains using different methods. In a study on Trolox (6-hydroxy-2,5,7,8-tetramethylchroman-2-carboxylic acid)-equivalent antioxidant capacity (TEAC), the free radical scavenging ability of betalains from red beets was found to be 1.5–2.0 times higher than that of anthocyanins at a pH above 4 [32]. With pH 7.4 and DPPH assays, the betalains extracted from beetroots showed 7.5 and 3.0 times higher free radical scavenging activity than vitamin C, respectively [40]. Studies on the structure and antioxidant activity of betalains from various Amaranthaceae plants have demonstrated that glycosylation reduces their ability to scavenge free radicals, thereby reducing their antioxidant activity. The position of glycosylation also affected their free radical scavenging ability. For example, 6-O-glycosylated betacyanins showed more powerful activity than 5-O-glycosylated betacyanins. In addition, this activity was also correlated with the number of hydroxyl/imino groups in betalains. The presence of catechol appears to be particularly important for the free radical scavenging properties of betanins. Betaxanthins have only a moderate free radical scavenging activity as they contain no phenolic hydroxyl groups (Figure 4). In general, betacyanins display more potent antioxidant activity than betaxanthins [40,41,42].

The quality of plant extracts depends on many factors, including the state of the original plant (type, part, and maturity), geographical origin, climatic conditions, harvest date, and storage. Furthermore, environmental factors and processing techniques also affect the quality of extracts [22,33]. Bucur et al. extracted betalains from beets harvested in either spring or fall and found that the betalain content in fresh spring beets was up to 8.38 ± 1.14 mg/g, nearly two times higher than that of fresh fall beets (4.62 ± 0.18 mg/g) [33]. The betalain content in frozen fall beets was found to be merely 2.9 ± 0.16 mg/g. In this study, the antioxidant activity of betalains was measured by the DPPH scavenging rate. The results showed that DPPH scavenging ability was positively correlated with the betalain content, as well as the storage method and harvest season [43]. Gliszczynska-Swiglo et al. cleaned, peeled, and squeezed the beetroot, acidified the juice with 1 N HCl to pH 3.0, left it at 4 °C overnight, and collected the supernatant by centrifugation. Betalains were then further separated from the juice by gel filtration using Sephadex G-25 chromatography (40 × 2.2 cm) [44]. The antioxidant activity of isolated betalains was then assessed by a modified TEAC method. This study revealed that the free radical scavenging activity of betalains is abnormally high at a pH above 4, especially in neutral and alkaline solutions. This was 1.5–2.0 times higher than those of ordinary anthocyanins. This result was attributed to the mono-, di-, and tri-deprotonated structures of betalains that showed stronger hydrogen- and electron-donating abilities in high pH conditions. In a study on the antioxidant activity of beet juice–gum arabic microcapsules prepared by spray drying, the changes in antioxidant activity were measured using DPPH and redox potential assays [45]. The results showed that the microcapsules had the best stability when *a*_w_ < 0.521; a longer storage time and higher water activity (*a*_w_ > 0.748) were observed. Notably, the antioxidant and radical-scavenging abilities of these microcapsules increased with decreasing stability. It has also been shown that when betaxanthin is thermally degraded to 60% of its original content, its total phenolic content and antioxidant activity slightly increase [46]. Given that betaxanthin can be degraded into a phenolic compound with higher antioxidant activity than its original precursor via beet proteases, the degradation product may possess enhanced antioxidant properties.

#### 3.1.2. Antioxidant-Related Effects of Betalains

Protection against lipid peroxidation remains one of the essential requirements for antioxidant performance in vivo [47]. Preliminary data obtained from in vitro studies on liposome oxidation suggested that both betanin and betanidin possess inhibitory effects against the peroxidation of cytochrome c and lipid, low-density lipoprotein (LDL) oxidation, and heme breakdown at very low concentrations [48,49]. These compounds also showed a nitric oxide-scavenging ability [50]. In a study by Allegra, betanin showed an improved inhibitory effect against the peroxynitrite-dependent nitrification of tyrosine compared to ascorbic acid, showing IC50 values of 19.2 µM and 79.6 µM, respectively [51]. It also inhibited the production of lipid hydroperoxides in human LDL under MPO/nitrite-induced oxidation. Betanin, at concentrations ranging from 0.05 to 1.0 mM, inhibited nitrite-induced DNA strand cleavage in a concentration-dependent manner and scavenged nitrogen dioxide, acting as a lipid peroxyl radical scavenger. Betanidin protects cells from oxidative and nitrative stresses [52]. Betanin and betaxanthin have been found to be good electron donors for myeloperoxidase (MPO) compounds I and II, which can interfere with the catalytic cycle of MPO and enhance its chlorination activity at very low concentrations and pH 7.0 [53]. The greatest release of HOCl from MPO can be reached at pH 5.0, and these two types of betalains showed scavenging effects at low micromolar concentrations. In addition to scavenging free radicals, betalains exert protection against LDL oxidation in vivo, presumably through the transactivation of paraoxonase 1 (PON1), which is an antioxidant enzyme produced in the liver [47,54].

### 3.2. Chemoprevention and Anti-Cancer Effects

The anti-cancer properties of beetroot were first suggested by Hungarian physician A. Ferenczi, who used beetroot for cancer treatment in the early 1950s. Betalains, used as a component of anti-cancer drugs, has been patented in the United States (Patent No: US2002178399) [9,55]. Studies on its chemoprevention and anti-cancer effects are summarized in Table 2.

Carcinogenesis refers to the transformation of normal cells into tumor cells, a process that involves three major phases (initiation, progression, and development). What drives these phases is oxidative stress and inflammation, which, in turn, cause a myriad of aberrant gene expressions in the transformed cell population and surrounding cells of the lesion [60]. By normalizing these inappropriate gene activities, bioreactive foods or their extracted or purified components can chemically prevent cancer. The molecular mechanism of the chemoprevention effects of betanin has not yet been elucidated. However, it has been observed that betanin neutralizes the inflammation induced by the release of reactive oxygen species (ROS) and hypochlorous acid (HClO) from neutrophils, thereby reducing oxidative stress and inhibiting the formation of new blood vessels in tumors [61,62]. These observations suggest that one of the inhibitory mechanisms of betanin involves the negative regulation of the development of stromal elements in inflammatory lesions. Betanin is non-mutagenic and affects transformed cells mainly by reducing their growth rate and inducing apoptosis [62,63]. Thus, betanin affects both the tumor microenvironment and cancer cells.

### 3.3. Prevention of Cardiovascular Diseases

Rahimi et al. investigated the effects of betalains on the expression of sirtuin-1 (SIRT1) and lectin-like oxidized low-density lipoprotein receptor 1 (LOX1) in patients with coronary artery disease [64]. SIRT1 is a longevity factor that regulates the human cell lifespan and affects various aspects of age-dependent atherosclerosis. The activation of SIRT1 is considered an effective way to prevent cardiovascular disease. In this study, patients with coronary artery disease ate a beetroot supplement rich in betalains once a day for two consecutive weeks. The subsequent analyses showed that the level of SIRT1 significantly increased in these patients, reaching a 2.67 times higher level than that of the placebo group [64]. Furthermore, the patients showed markedly decreased levels of LOX1 and the plasma high-sensitivity C-reactive protein (HS-CRP). These results indicate that betanidin has a preventive effect against age-related diseases. In another study, blood and urine samples were collected from male patients with acute coronary atherosclerosis at 3, 8, and 24 h after supplementation with 50 mg of betanin [64]. HPLC analysis showed that the concentrations of betanin in plasma and urine were 0.13% and 0.93%, respectively, along with significantly reduced concentrations of homocysteine, glucose, total cholesterol, triglycerides, and low-density lipoproteins [65]. In addition, supplements rich in betalains have been demonstrated to reduce systolic and diastolic blood pressure.

### 3.4. Improvements in Athletic Performance

Betalain is an indole pigment that may have an energizing effect, as reported in recent studies. Mumford et al. conducted a circulation time test (TT) on male cyclists (29 ± 10 years old, 77.3 ± 13.3 kg, 3.03 ± 0.62 W/kg) who were supplemented with 100 mg of betanin-rich beetroot concentrate (BLN) every day for 7 days before, during, or after the TT [66]. The evaluation indicated that the circulatory performance and hemodynamics in the experimental group were improved. Specifically, compared to the placebo group, the experimental group displayed an elevated mean absolute power, a significantly improved exercise efficiency 5 min after TT, and a greater brachial artery blood flow immediately following exercise. These results support the hypothesis that BLN may have potential benefits for competitive athletes. Likewise, Petrie et al. [67] showed that the combination of exercise and beet juice intake can enhance the potential neuroplasticity of the elderly. Beet juice has been demonstrated to increase blood flow to the brain and improve exercise performance.

### 3.5. Pharmaceutical Industries

The pharmaceutical industry has long been exploring natural resources to develop new drugs and therapies. Sugar beets, not only a nutritional supplement in our diet, also have significant potential in the medical field. The effective application of sugar beets in the pharmaceutical industry demonstrates their versatility and rich composition of bioactive compounds. They are rich in nitrates, which are converted into nitric oxide in the body. Nitric oxide helps relax and dilate blood vessels, thereby lowering blood pressure and improving blood flow. This characteristic makes beetroot extract a valuable ingredient in drug formulations for the prevention and treatment of hypertension and other heart-related diseases [68]. In addition, sugar beets are rich in antioxidants, such as betacyanins, which have been shown to combat oxidative stress and inflammation in many chronic diseases, such as arthritis and diabetes [69]. Sugar beets are also a source of betaine, a compound related to liver health. Betaine helps protect cells, proteins, and enzymes from environmental stress and has been used in the treatment of liver diseases such as nonalcoholic fatty liver disease (NAFLD) [70]. In the field of oncology, sugar beets are being studied for their potential role in cancer prevention and treatment. The betacyanins found in sugar beets have shown anti-cancer properties in various laboratory studies. They are believed to inhibit the growth of cancer cells in certain types of cancer and induce apoptosis or programmed cell death [71]. Another interesting aspect is the potential of sugar beets in neurology. The nitrates in sugar beets are associated with improved cognitive function and may have protective effects on the brain. This has led to investigations into the possible use of beetroot extract as a form of treatment for neurodegenerative diseases such as Alzheimer’s disease and Parkinson’s disease [72].

## 4. Other Bioactive Substances

### 4.1. Dietary Fiber

Plants generally contain considerable amounts of dietary fiber, which promotes the development and protection of beneficial intestinal flora. In a recent study, the dietary fiber content in wild Swiss beet leaves was determined by the AOAC enzyme-weight method [73]. This evaluation showed that wild beet leaves contain a total dietary fiber of 2.43 g per 100 g fresh weight, with insoluble dietary fiber (IDF) as the dominant fraction (2.30 g IDF/2.43 g total). This content is higher than the total dietary fiber found in cultivated beet leaves (1.6 g/100 g fresh weight, US Department of Agriculture). Nishimura et al. prepared dietary fiber samples from beets and examined their effects on plasma cholesterol in rats [74]. The results showed that after 21 days of supplementation, the groups fed with 5% or 10% dietary fiber added to the feed showed a significantly reduced plasma cholesterol content, body weight, and liver cholesterol content compared to the group fed with a regular diet [74]. In an earlier study, Hara reported a similar result, suggesting that the cecal and colonic fermentation of dietary fiber is related to fiber’s cholesterol-lowering effect [75]. Elevated blood cholesterol levels are considered a risk factor for coronary heart disease. The dietary fiber in beets can help prevent these diseases by regulating plasma cholesterol concentrations.

### 4.2. Fatty Acids

Mzoughi et al. conducted a chemical screening of Swiss beet leaves and found that they contain fatty acids, such as stearic acid, palmitic acid, linoleic acid, and linolenic acid, with linoleic acid having the highest content, followed by a significantly high level of oleic acid [60]. Both linoleic acid and oleic acid have been shown to play important roles in the construction of nerve cells. Among saturated fatty acids, palmitic acid is predominant (22.92%). It has also been demonstrated that Swiss beet leaf oil is rich in unsaturated fatty acids and has a fundamental role in the prevention of cardiovascular disease. A significant unsaturated/saturated ratio (U/S) is considered beneficial for reducing serum cholesterol and atherosclerosis, as well as preventing heart disease [76].

### 4.3. Volatile Compounds

Using a Varian CP3800 gas chromatograph equipped with a DB-5 capillary column (30 mm–0.25 mm–0.25 µm, InertCap WaX, Agilent, Hong Kong) and a Varian Saturn 2000 ion trap mass detector (Varian, Walton-on Thames, UK), a total of 36 volatile components were identified in Swiss beet leaves, accounting for 95.1% of the total aroma [73]. Quantitative analysis showed that non-terpene derivatives (mainly including linear aldehydes and alcohols) and oxygen-containing monoterpene alcohols, ketones, phenols, and ethers are the main volatile components of beet leaves, accounting for 40.6% and 27.7%, respectively. In addition, phenylpropanoid is also highly present in the leaves, accounting for 11.3%. It has been reported that these compounds have various biological activities, including antibacterial, antioxidant, anti-inflammatory, anti-diabetic, anti-cancer, kidney-protective, neuroprotective, cardiac-protective, and liver-protective effects (as shown in Table 3) [77]. In contrast, sesquiterpene hydrocarbons are hardly detected in the leaves (2.0%). The main volatile components of beet leaves include (E)-anethole (11.3%), caprylic acid (7.5%), decanal (7.3%), α-terpineol (5.8%), and limonene (5.6%). Among these compounds, anethole has shown powerful antibacterial properties, particularly against bacteria, yeast, and fungi [78].

While the compositional properties of volatiles in Tunisian beet leaves are similar to those in Swiss beet leaves, the proportions of the components vary between the two types. Zardi Bergaoui et al. first used Gas Chromatography with Flame Ionization Detection (GC-FID) (Burk scientific M910) and Gas Chromatography–Mass Spectrometry (GC-MS)) (QP2020 Shimadzu, Kyoto, Japan) to determine the chemical composition of volatile oils in Tunisian beet leaves, with a yield of 0.037% (*w*/*w*), and identified a total of 25 components, which accounted for 98.1% of the total oil content [79]. The volatile oil was mainly composed of oxygen-containing sesquiterpenes (39.2%), followed by sesquiterpenoid hydrocarbons (30.3%) and rotenoid (26.3%). In addition, the main compounds of volatile oil included γ-ferricone (26.3%), α-terpineol (12.1%), T-terpineol (10.6%), bicyclic macrocaryophyllene (10.4%), and δ-junitol (6.0%). In this study, the antioxidant activity of the separated oil was evaluated by DPPH, ABTS+, catalase, and paraoxonase assays. The oil’s cytotoxicity, anticholinesterase activity, and anti-tyrosinase activity were also evaluated. The results indicated potent antioxidant (IC50: 0.055 ± 0.006 mg/mL) and catalase activities (524.447 ± 2.58 units/mg protein), along with significant cytotoxicity against A549 cells (IC50: 42.44 ± 1.40 μg/mL).

## 5. Conclusions and Perspectives

Beets (*Beta vulgaris* L.), one of the first crops developed based on modern genetic principles, are a typical halophyte with high amounts of secondary metabolites (e.g., phenolic compounds synthesized under stress conditions, such as ultraviolet light and salt stress tolerance) and high biological activity. Traditionally, beets have been used in folk remedies to treat kidney and liver disorders due to their beneficial health effects. They can stimulate the immune and hematopoietic systems and act as a unique dietary treatment for cancer. Phytochemicals in beets exhibit complementary mechanisms of action in cancer prevention, presumably by scavenging oxidants, regulating gene expression in cell proliferation, and modulating detoxification enzymes. Therefore, with the increasing attention to the nutritional and pharmacological potential of beets, the systematic extraction, isolation, and characterization of the chemical components of their leaves, stems, and roots, as well as the determination of their functional activities and their synergistic mechanism among various functional components, could provide a theoretical basis for their further application in health food and medicine.

## Figures and Tables

**Table 1:** The phenolic content of different beet parts, dividing by phenol type and beet type.

Beet Type	Phenol Type	Different Beet Parts	Phenolic Content (mg/g)	Reference
Leaf feet	Ferulic acid derivatives, feruloyl glucosides a and b, quercetin pentoside, coumaric acid derivatives, apigenin glucoside, quinic acid derivatives, etc.	Leaf	25~35	Calvo [13]
		Petiole	3~5	Maravić [14]
Table beets	Betaine dihydroflavone, xanthophyll A, dihydroisorhamnetin, saponins, vanillic acid, chlorogenic acid, syringic acid, etc.	Beetroot	0.8~2	Vasconcellos [15]
		Beetroot slices	0.5~1.2	Kavalcová [16]
Fodder beet	Apigenin, syringic acid, kaempferol, chlorogenic acid	Beetroot slices	0.4~0.8	Kavalcová [17]
		Petiole	0.8~2.5	Bangar [18]
Sugar beet	Ferulic acid, chlorogenic acid, caffeic acid, vanillic acid, p-coumaric acid, isorhamnetin-3-O-glucoside	Petiole	10~20	Liu [19]
		Beetroot	5~10	Smith [20]

**Table 2:** Chemoprevention and anti-cancer effects of betalains.

Chemoprevention and Anti-Cancer Effects	Experimental Approaches	Experimental Results	Reference
Liver protection and anti-cancer effects	Non-tumor THLE-2 and liver cancer HepG2 cells were used to investigate the effects of betanin on the activation of nuclear factor erythroid-2-related factor 2 (Nrf2), as well as on the expression of GSTA, GSTP, GSTM, GSTT, NQO1, and HO-1.	Betanin induced Nrf2-controlled gene expression, presumably by activating Nrf2.	Krajka-Kuz’niak [56]
In vivo antioxidant and potential hepatoprotective properties	The in vivo antioxidant and potential hepatoprotective properties of betanin were evaluated by assaying the activities of several enzymes (xanthine oxidase, catalase—CAT, peroxidase, glutathione peroxidase—GSHPx, and glutathione reductase), as well as the levels of glutathione-GSH and glutathione-barbituric acid reactive substances (TBARS) in Wistar rats fed for 7 to 8 weeks.	Betalains showed antioxidant and hepatoprotective effects.	Jelena J. Vulic [57]
Inhibition of breast cancer cell (MCF-7) proliferation	The expression of apoptosis-related proteins (Bad, TRAILR4, FAS, and p53) and changes in mitochondrial membrane potential in MCF-7 cells were analyzed to investigate the involvement of betanin/isobetanin in the internal and external apoptotic pathways of cells.	Betanin/isobetanin concentrate significantly reduced cancer cell proliferation and viability.	Nowacki [58]
Inhibition of prostate cancer cell growth	The roles of betalains in DU-145 and PC-3 prostate cancer cells were examined, including inhibitory effects against cell proliferation, cell migration, colony formation, and cell growth-related signaling pathways.	In PC-3 cells, betalains showed no regulatory effects on the proteins of the mTOR pathway but decreased the phosphorylation level of S6K1 and increased the content of the mTOR protein. In DU-145 cell assays, betalains exerted a regulatory effect on the mTOR pathway, reducing the mTOR level and total content of mTOR. In terms of apoptosis and cell cycle pathways, 100 μg/mL of betalain could significantly reduce Bcl-XL levels and the PARP1 content in DU-145 cells.	Mancini [27]
Chemoprevention against colon cancer	The cytotoxicity of glucosinolate-2-O-xyloside (XVX) (derived from beet seeds), betaxanthin (R1), and betacyanins (R2) against CaCo-2 colon cancer cells was examined.	The combination of R2 and R1 with XVX could significantly prolong the cytotoxicity of the latter; this effect was mediated through the intrinsic apoptosis pathway. R1 and R2, either alone or in combination, could reduce H_2_O_2_-triggered oxidative stress in CaCo-2 cells. These results suggest that R1, R2, and XVX can serve as a chemopreventive agent for colon cancer.	Farabegoli [59]

**Table 3:** Various phenylpropanoid derivatives along with different biological activities and their effects.

Compound name	Compound/Structure	Activity	Effects
CiA derivative	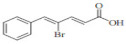	Antioxidant activity	Scavenges hydroxyl radicals and inhibits linoleic acid peroxidation and soybean LOX activity petiole
Phenylpropanoids derivatives-11	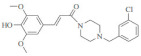	Antioxidant and anti-inflammatory activity	Intracellular ROS and ear edema. Inhibited TNF- a-induced NF-jB activation, ICAM-1, and VCAM-1 expression in ECs
N-trans-feruloyldopamine	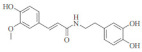	Antioxidant and neuroprotective activity	Scavenges free radical and chelates ferrous ions Inhibited AChE activity
Ferulic acid-O-alkylamine derivative,	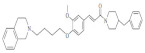	Neuroprotective activity	Inhibited BuChE and AChE activity. Prevented selffibrils from aggregation and induced Ab1-42, promoting its disaggregation
Cinnamic acyl 1,3,4-thiadiazole amide derivatives	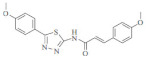	Anti-cancer activity	Inhibited tubulin polymerization and the anti-proliferation of A549 and MCF-7 cell lines
4-methoxy cinnamic acid	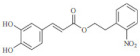	Cardioprotective activity	ROS, cardiac inflammation, collagen deposition, and NF-jB activation. Stimulated (SIRT-1)/eNOS expression. Reduced necrocytosis and cell apoptosis

## Data Availability

Not applicable.
